# Dosimetric impact of inter-observer variability for 3D conformal radiotherapy and volumetric modulated arc therapy: the rectal tumor target definition case

**DOI:** 10.1186/1748-717X-8-176

**Published:** 2013-07-09

**Authors:** Francesca Lobefalo, Mario Bignardi, Giacomo Reggiori, Angelo Tozzi, Stefano Tomatis, Filippo Alongi, Antonella Fogliata, Anna Gaudino, Piera Navarria, Luca Cozzi, Marta Scorsetti, Pietro Mancosu

**Affiliations:** 1 Radiation Oncology Department, Humanitas Clinical and Research Center, Rozzano, Milan, Italy; 2 Poliambulanza Foundation Hospital, Radiation Oncology, Brescia, Italy; 3 Oncology Institute of Southern Switzerland, Medical Physics Unit, Bellinzona, Switzerland

**Keywords:** Target definition, Intervariability, Dosimetry, Volumetric modulated arc therapy, RapidArc, 3D Conformal radiotherapy, Rectal cancer

## Abstract

**Background:**

To assess the dosimetric effect induced by inter-observer variability in target definition for 3D-conformal RT (3DCRT) and volumetric modulated arc therapy by RapidArc (RA) techniques for rectal cancer treatment.

**Methods:**

Ten patients with rectal cancer subjected to neo-adjuvant RT were randomly selected from the internal database. Four radiation oncologists independently contoured the clinical target volume (CTV) in blind mode. Planning target volume (PTV) was defined as CTV + 7 mm in the three directions. Afterwards, shared guidelines between radiation oncologists were introduced to give general criteria for the contouring of rectal target and the four radiation oncologists defined new CTV following the guidelines. For each patient, six intersections (I) and unions (U) volumes were calculated coupling the contours of the various oncologists. This was repeated for the contours drawn after the guidelines. Agreement Index (AI = I/U) was calculated pre and post guidelines. Two RT plans (one with 3DCRT technique using 3–4 fields and one with RA using a single modulated arc) were optimized on each radiation oncologist’s PTV. For each plan the PTV volume receiving at least 95% of the prescribed dose (PTV V95%) was calculated for both target and non-target PTVs.

**Results:**

The inter-operator AI pre-guidelines was 0.57 and was increased up to 0.69 post-guidelines. The maximum volume difference between the various CTV couples, drawn for each patient, passed from 380 ± 147 cm^3^ to 137 ± 83 cm^3^ after the introduction of guidelines. The mean percentage for the non-target PTV V95% was 93.7 ± 9.2% before and 96.6 ± 4.9%after the introduction of guidelines for the 3DCRT, for RA the increase was more relevant, passing from 86.5 ± 13.8% (pre) to 94.5 ± 7.5% (post). The OARs were maximally spared with VMAT technique while the variability between pre and post guidelines was not relevant in both techniques.

**Conclusions:**

The contouring inter-observer variability has dosimetric effects in the PTV coverage. The introduction of guidelines increases the dosimetric consistency for both techniques, with greater improvements for RA technique.

## Background

Modern radiation therapy techniques with inverse planning optimization are able to achieve optimal dose painting covering any desired volume. In this context accurate target delineation is vitally important to ensure that the target is not under-treated and to limit the dose to surrounding normal tissues. At this purpose, recent reports recommended the creation of a target definition consensus and stated the importance of specific educational interventions concerning target contouring.

Pre-operative chemo-radiotherapy of rectal cancer in locally advanced stage has become a widely accepted treatment modality. Locally advanced rectal cancer treated with neo-adjuvant chemoradiation therapy is expected: a) to show positive response with tumour down-staging in about half of patients [1]; b) to obtain better results in terms of local control compared to adjuvant approach as shown in a phase III study [2]. The new technologies in radiotherapy, such as intensity modulated radiotherapy (IMRT) or more recently volumetric modulated arc therapy (VMAT), allow to achieve highly conformed dose distribution on the target volume and to spare the adjacent healthy tissues (HT) and organs at risk (OAR). In several studies with patients receiving pelvic irradiation for rectal or anal cancer, it has been shown that IMRT and VMAT are dosimetrically superior to other conformal techniques in protecting normal tissue close to the target [3]. Roberton et al. [4] showed dose-volume relationship between bowel irradiation and acute grade 3 diarrhoea to be clearly correlated and suggested the need of reducing as much as possible the OARs involved in preoperative irradiation of rectal cancer. Thus a contouring methodology shared by the group is a fundamental topic, as assessed by many works on rectal cancer [5-8].

In this paper we have investigated the dosimetric impact of introducing educational interventions in the delineation of the rectal target. We conducted a study in which participating radiation oncologists delineated target contours before and after the introduction of shared guidelines. The aim of the study was to evaluate and compare the dosimetric effects of target contouring variability in cases of 3Dconformal RT (3DCRT) and of RapidArc (RA) techniques. Plans were optimized for each target delineated by the radiation oncologists. The primary endpoint was to evaluate the dosimetric coverage of the remaining radiation oncologist’s targets defined on the same patient. Secondary endpoint was the evaluation of doses at OAR for the two techniques. The contouring inter-observer variability within the radiation oncologists of the group before and after the introduction of shared guidelines was preliminarily evaluated.

## Methods

### Patient selection

Ten patients (seven males and three females) with pathologically proven rectal cancer in locally advanced stage, subjected to neo-adjuvant RT with curative intent were considered in the present analysis. Patients were randomly selected from the internal database of patients; to avoid possible biases in contouring, the patients’ names were hidden and associated with a progressive numeration. Computed tomography (CT) datasets were acquired with a 3-mm slice thickness from a 16 slice CT system, in free breathing condition. Patients, with arms raised above the neck, were in prone position and immobilized with Belly-Board devices to dislocate anteriorly as much as possible intestinal loops of small bowel.

Four radiation oncologists were involved in this study. Each of them was asked to contour the clinical target volume (CTV) for each of the ten patients in blind mode (i.e. radiation oncologists could not see the contours of the other oncologists involved in the study). After that, our institute’s rectal cancer referential radiation oncologist established a consensus-based guideline on CTV delineation, in order to share some general criteria for the contouring of rectal target. After a minimum of one month, the same four radiation oncologists contoured the ten targets following the guidelines, in blind mode, too (i.e. radiation oncologist’s could not see neither the other physician’s contours nor their own previous ones).

The planning target volume (PTV) was defined adding three-dimensional 7 mm margins to the CTV.

### Target definition guidelines

This educational intervention included a formal guideline, available on-line in our department, as well as an initial teaching session involving all physicians taking part to this study. The CTV had to include the entire mesorectum, the presacral and internal iliac nodal regions, the gross tumor with a cranial and caudal margin of at least 2 cm. Criteria for CTV delineation strictly followed guidelines from Roels et al. [6]. Since RTOG atlases are commonly used in our department by means of on-line links at the contouring workstations, participants were carefully informed about differences of our criteria compared with those in the RTOG paper from Myerson et al. [5].

### Planning techniques

For each patient, 8 plans (4 3DCRT and 4 RA) were optimized before the introduction of the guidelines and other 8 were optimized after the guidelines introduction. Each PTV drawn by the four physicians before and after the guidelines introduction was set as plan target of one of the 16 plans optimized for each patient. A standard protocol was adopted for all plans: dose prescription was set to 50.4 Gy to mean PTV in fractions of 1.8 Gy/day. For all PTVs, plans aimed to achieve V_95%_ > 95% (at least 95% of the PTV volume must be covered by 95% of the prescribed dose) and a maximum dose (i.e. D_2%_ as defined in ICRU 83) lower than 107%. Bowel (defined as the entire peritoneal cavity), bladder and femoral heads were considered as OARs. The mean dose, maximum dose (D_2%_) and appropriate values of V × Gy (volume receiving at last × Gy) were scored. Planning objectives for OARs were defined as follows: bowel V_45Gy_ < 80 cm^3^ and V_50Gy_ < 20 cm^3^; no hotspot inside the bladder was allowed, D_30%_ < 35 Gy, and mean dose objective was <45 Gy; femoral heads maximum dose (D_2%_) < 47 Gy [3]. The planning objectives for HT were not numerically formalised, the strategy was to minimise its involvement.

The 3DCRT series were planned according to our institute’s practice with three fields (one posterior and two laterals with wedges) or four fields (posterior, anterior, and two laterals with wedges) with 6 MV or 18 MV energy. The beam arrangement was set in order to obtain the best solution according to the target shape. Conformal shaping of the fields was performed by means of static MLC, setting 5 mm MLC margin in lateral and 7 mm in cranial-caudal direction. The RA plans consisted of a single 360° arc of 6 MV; the RA plans were optimized starting with a common dose volume histogram (DVH) objective template. All plans were normalized to the mean dose of the target PTV (i.e.100% at target mean). Both techniques were optimized using Varian Eclipse treatment planning system (version 8.9) on a 2100-DHX Varian Linac, equipped with a Millennium MLC (leaf width at isocentre of 5 mm in the central 20 cm part of the field, 10 mm in the outer 2 × 10 cm and a leaf transmission of 1.7%). All dose distributions were computed with the Anisotropic Analytical Algorithm (AAA) implemented in the Eclipse planning system with a calculation grid resolution of 2.5 mm.

### Data analysis

Firstly, the contours were evaluated from the geometrical point of view. In particular, for each patient the CTV volumes were measured and the variation was calculated as the maximum volume difference between two CTVs among the four targets (one for each physician) drawn on a same patient. The percentage volume variation was calculated for each patient’s target, defined as Δ = 100 × (*V*_*max*_–*V*_*min*_)/*V*_*mean*_. Furthermore the ratio (*V*_*max*_/*V*_*min*_) was reported. These definitions were used to give information about the deviation regardless of the volume absolute values. Concerning the interobserver contouring variability, for each patient six intersections (I) and unions (U) volumes were calculated coupling the contours of the various oncologists. This was repeated for the contours drawn after the guidelines. Agreement Index (AI) (i.e. with V_i_ and V_j_ the volume delineated by the i-th and j-th physician) $AI_{\mathrm{ij}}=\frac{V_{i}\cap V_{j}}{V_{i}\cup V_{j}}$ was calculated for each target and for all possible couples of contours pre and post guidelines introduction.

**Figure 1:**
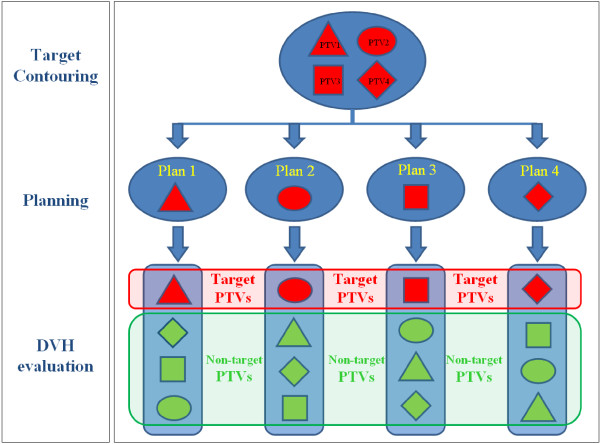
**A schematic representation of the PTV analysis is presented: the four physicians defined four different targets on the same patient, for each patient four plans were optimized on the different PTVs.** During the DVH evaluation the PTV on which the target was optimized was called *target PTV*, while the other targets present on the same CT series were called *non*-*target PTVs*. Obviously all targets were both *target* and *non*-*target PTVs* according to the plan considered. This procedure was performed for each patient and for the two planning techniques (3DCRT and RA) before and after the introduction of the guidelines.

Quantitative evaluation of plans was performed by means of DVH. For PTV the following data were reported and used as a parameter: target coverage evaluating the PTV V95%: PTV’s volume receiving at least 95% of the prescribed dose (dose prescription: 50.4 Gy).These parameters were evaluated separately for the target PTV (i.e. the PTV on which the plan was optimized) and for non-target PTVs (see Figure 1) in order to assess the dosimetric impact of the target definition uncertainty for both the techniques considered. This analysis was performed before and after the introduction of guidelines in order to evaluate a possible dosimetric improvement.

### Study design and statistical analysis

The present study was performed as part of the internal quality process for improving RT practice. Ten CT scans were considered as a representative sample of the procedure. Contours and plans were compared with the Wilcoxon matched-pair signed-rank test for non-parametrically distributed data. The threshold for statistical significance was set at p < 0.05. The analysis was performed using Statistica 6.0 (Vigonza, Italy).

## Results

### Contouring inter-observer variability

**Figure 2:**
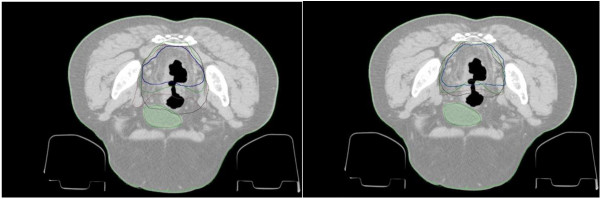
Four different contours of CTVs superimposed on a axial section of a CT image pre (left) and post (right) the introduction of guidelines.

A total of 80 contours were generated and analyzed. Each contour was superimposed on the original CT images. An example of target contouring drawn before and after the introduction of guidelines by the four radiation oncologists is shown in Figure 2.

Tables 1 and 2 report the analysis of the CTV volumes contoured before and after the guidelines introduction. In detail, the mean inter-operator variability, evaluated on CTV contouring, was evaluated before and after the introduction of the guidelines. For the pre-guidelines contours, mean CTV volume was 380 cm^3^ ranging from a maximum of 682 cm^3^ (patient 2) to a minimum of 117 cm^3^ (patient 7) and the mean value decreased to 137 cm^3^ ranging from 283 cm^3^ (patient 10) to 31 cm^3^ (patient 4) for the post guidelines contours. The ratio of the largest to the smallest contoured volume was 1.79 before and 1.27 after the introduction of guidelines. The inter-operator AI passed from 0.57 to 0.69 thanks to the guidelines introduction. The intra-observer AI before and after the guidelines introduction was 0.74, with significative target volume reduction.

**Table 1:** Analysis of the CTVs volume contouring pre and post the introduction of guidelines

***PRE***-***Guidelines***					
***Patient***	***Mean Volume *(*cm***^***3***^**)**	***Vmax*-*Vmin *(*cm***^***3***^**)**	***Range *[ *min *; *max*] (*cm***^***3***^**)**	**Δ (%)**	***Vmax*/*Vmin***
1	745	336	[656; 992]	45	1.51
2	772	682	[528; 1210]	81	2.08
3	545	494	[380; 874]	91	2.30
4	499	467	[366; 833]	94	2.28
5	540	302	[415; 716]	56	1.73
6	463	248	[330; 578]	54	1.75
7	559	117	[494; 611]	21	1.24
8	722	307	[618; 925]	43	1.50
9	648	480	[486; 966]	74	1.99
10	994	423	[790; 1213]	43	1.54
** Mean**	**649**	**380**	-	**60**	**1**.**79**
** St Dev**	**162**	**147**	-	**24**	**0**.**36**
** Median**	**603**	**380**	-	**55**	**1**.**74**
***POST*-*Guidelines***					
***Patient***	***Mean Volume *(*cm***^***3***^**)**	***Vmax*-*Vmin *(*cm***^***3***^**)**	***Range *[ *min *; *max*] ( *cm *)**	**Δ(%)**	***Vmax*/*Vmin***
1	797	98	[848; 750]	12	1.13
2	666	254	[771; 518]	38	1.49
3	496	136	[565; 429]	27	1.32
4	400	31	[417; 386]	8	1.08
5	483	40	[504; 465]	8	1.09
6	406	153	[483; 331]	38	1.46
7	502	168	[574; 406]	33	1.41
8	608	79	[642; 564]	13	1.14
9	574	132	[635; 503]	23	1.26
10	1019	283	[1174; 891]	28	1.32
** Mean**	**595**	**137**	-	**23**	**1**.**27**
** St Dev**	**192**	**83**	-	**12**	**0**.**15**
** Median**	**538**	**134**	-	**25**	**1**.**29**

**Table 2:** Results from the analysis of the Agreement Index

***Agreement Index *( *I */ *U *)_ *Pre *-*Guidelines***							
	***I*_ *Tot */ *U *_*Tot***	***1 vs 2***	***1 vs 3***	***1 vs 4***	***2 vs 3***	***2 vs 4***	***3 vs 4***
Mean;	0.39	0.60	0.62	0.66	0.58	0.55	0.60
[min; max]	[0.27; 0.51]	[0.48; 0.76]	[0.44; 0.73]	[0.53; 0.78]	[0.44; 0.70]	[0.42; 0.65]	[0.50; 0.74]
St. Dev	0.08	0.08	0.10	0.07	0.08	0.08	0.09
Err%	20.7	13.6	15.9	10.2	14.0	14.1	14.2
***Agreement Index *( *I */ *U *)_ *Post *-*Guidelines***							
	***I*_ *Tot */ *U *_*Tot***	***1 vs 2***	***1 vs 3***	***1 vs 4***	***2 vs 3***	***2 vs 4***	***3 vs 4***
Mean;	0.64	0.65	0.78	0.76	0.63	0.64	0.75
[min; max]	[0.46; 0.75]	[0.53; 0.77]	[0.62; 0.86]	[0.58; 0.88]	[0.51; 0.77]	[0.58; 0.70]	[0.56; 0.88]
St. Dev	0.11	0.07	0.09	0.11	0.09	0.04	0.11
Err%	17.6	10.8	11.9	14.0	13.7	6.7	14.6

The most relevant discrepancy in terms of target definition regarded the bilaterally inclusion of external iliac nodes. This differences influenced the anterior posterior target volume, while in cranial caudal direction no relevant differences were found (<1 cm).

### Target coverage and dose homogeneity

**Figure 3:**
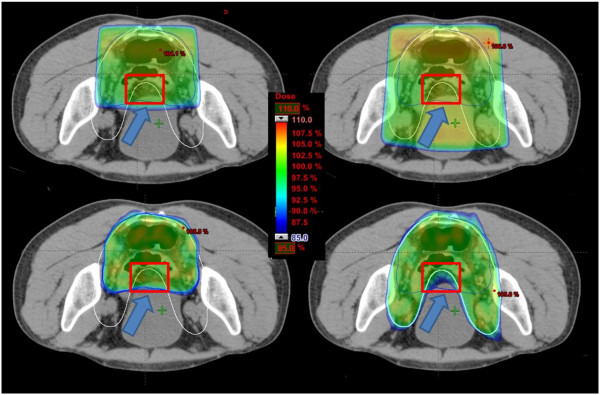
**Isodose distributions for an example patient for (up) 3D-CRT (low) RA optimized on PTVs defined by two physicians before the introduction of guidelines.** Doses are shown in colorwash ranging from 110% to 85% of the prescription dose. The arrows highlight an area (red rectangle in the figure) that is differently covered by 3D-CRT and RA. An under-dosage of the area is induced by the RA sculpting shape in comparison to the 3D-CRT’s coverage.

Figure 3 shows representative examples of dose distributions, using color-wash lookup table, obtained with both 3DCRT and RA techniques on the same patient. Plans on the left were optimized on the PTV defined by physicians 1 while plans on the right were optimized on the target drawn by physician 2 before the introduction of the guidelines. The target PTVs were fully covered both with 3D-CRT and RA though RA technique allows a better dose sculpting on the target and a dose reduction on neighbour HT. This dose sculpting, however, induces an under dosage on the non-target PTV (i.e. the PTVs delineated by the other physician on the same patient) for both plans (see arrows in Figure 3). On the contrary, the 3D-CRT, with the classical box approach, has a lower sparing of the neighbor tissue but allows a better coverage of the non-target PTVs, and only in one case inducing an under-dosage.

**Figure 4:**
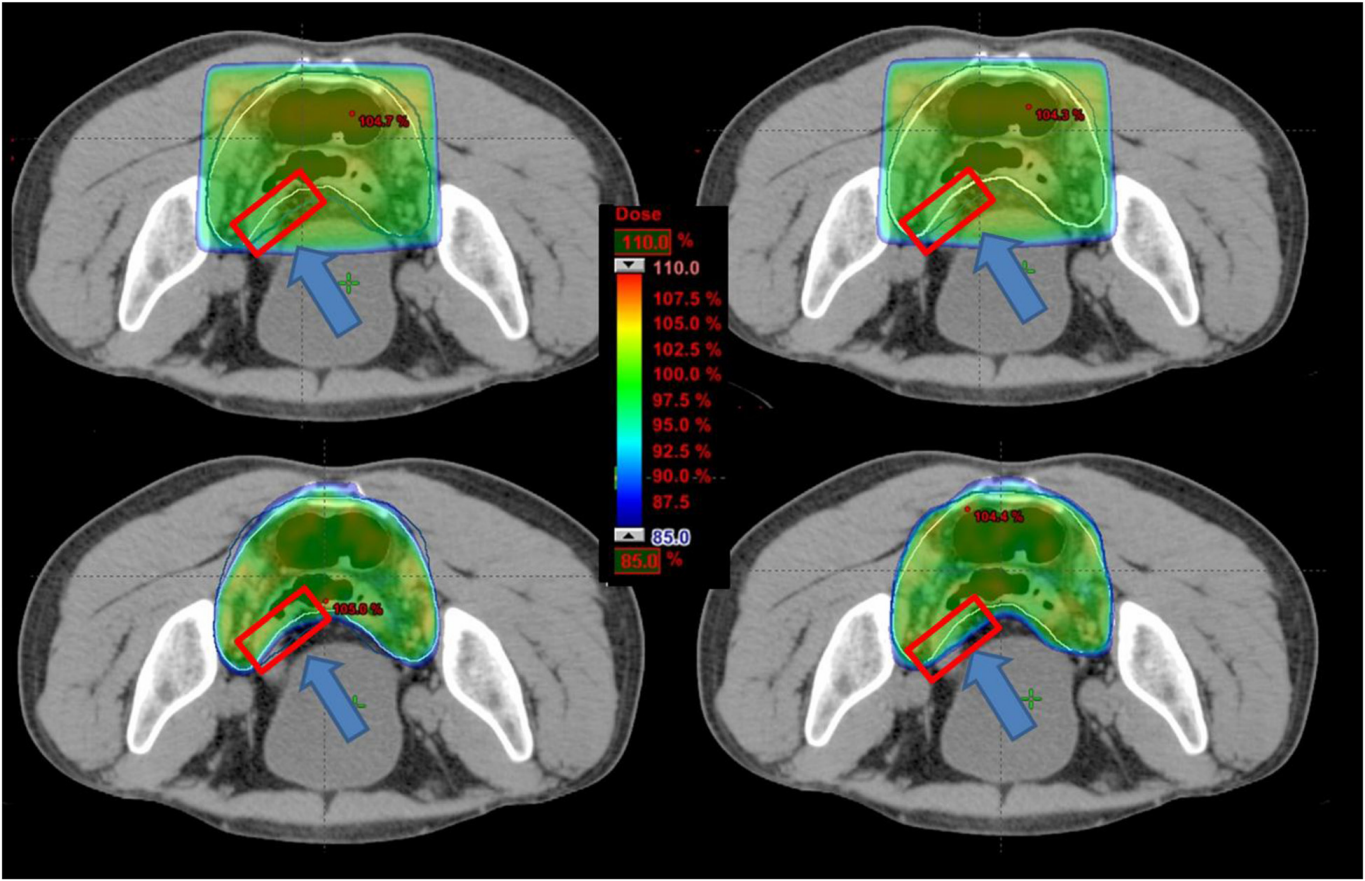
**Isodose distributions for an example patient for (up) 3D-CRT (low) RA optimized on PTVs defined by two physicians after the introduction of guidelines.** Doses are shown in colorwash ranging from 110% to 85% of prescription dose. The arrows highlight an area that is differently covered by 3D-CRT and RA. An under-dosage of the area is induced by the RA sculpting shape in comparison to the 3D-CRT’s coverage. To be noticed the underdosage area is lower than before the guidelines introduction.

Figure 4 shows the PTVs drawn by physicians 1 and 2 (the same of Figure 3) after the introduction of the guidelines. Dose distributions, using color-wash lookup table, are shown. As a consequence of the shared guidelines, the contours appear more similar and thus the dose distributions too; in this case, only a small area of the non-target PTV was not covered using the RA approach, while a complete coverage was met for the 3DCRT technique.

**Figure 5:**
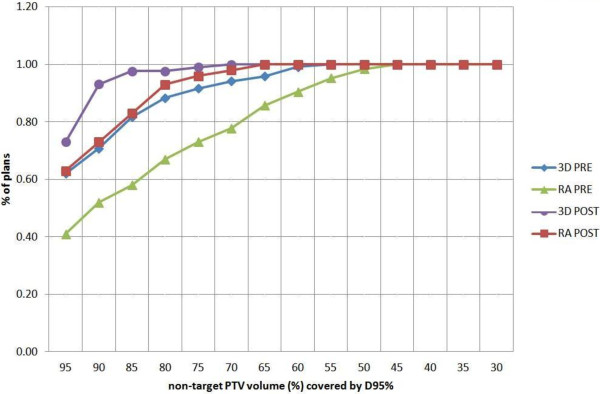
**Cumulative histogram representing the number of plans (%) with a certain non-target PTV volume fraction covered by the 95% isodose.** For example for 3D pre 83% of the plans had at least 85% of the non-target PTV volume covered by the 95% isodose.

Table 3 reports the systematic DVH analysis for the 3DCRT and RA techniques before and after the guidelines introduction. Data in the table are normalized to the prescription dose (100% corresponds to 50.4 Gy). In detail, the target PTV always fulfilled the objectives in terms of target coverage (95% of the volume received 95% of the prescribed dose). Considering the non-target PTVs, instead, the mean volume receiving 95% of the prescribed dose was 93.7 ± 9.2% before and 96.6 ± 4.9% after the introduction of guidelines for the 3DCRT; for RA plans the increase was more relevant, going from 86.5 ± 13.8% (pre) to 94.5 ± 7.5% (post). Furthermore the percentage of plans that had an acceptable non-target PTV coverage (i.e. V_95%_ ≥ 95%) passed from 62% to 73% (+11%) for 3D-CRT, while for VMAT plans the increase was +22% (from 41% to 63%) (see Figure 5).

**Table 3:** **Summery of DVH analysis for PTV, bladder and femurs**

	**3D-PRE**	**3D-POST**	**RA-PRE**	**RA-POST**	**p**
	**PTV**_**Target**_				
V_95%_ [%]	99.8 ± 0.2	99.9 ± 0.1	99.7 ± 0.2	99.7 ± 0.2	-
	**PTV **_**Non Target**_				
V_95%_ [%]	93.7 ± 9.2	96.6 ± 4.9	86.5 ± 13.8	94.5 ± 7.5	a,b,d,f
	**Bladder**				
D_2%_ [Gy]	48.5 ± 5.1	49.0 ± 5.2	47.4 ± 3.6	46.1 ± 4.2	-
Mean [Gy]	33.4 ± 8.5	30.1 ± 6.5	32.1 ± 5.7	30.6 ± 3.8	c
D_30%_ [Gy]	25.9 ± 5.4	18.3 ± 5.7	18.7 ± 3.3	17.6 ± 2.1	a,b,c
V_40Gy_ [%]	20.7 ± 12.5	14.9 ± 9.5	11.9 ± 10.1	8.1 ± 5.4	a,b,c,d,e,f
	**Right Femur**				
Mean [Gy]	32.0 ± 7.5	29.9 ± 6.6	19.7 ± 4.5	18.4 ± 1.9	b,c,d,e
D_2%_ [Gy]	41.7 ± 5.6	42.7 ± 4.7	28.2 ± 7.4	28.1 ± 3.9	b,c,d,e
	**Left Femur**				
Mean [Gy]	31.9 ± 7.0	30.2 ± 6.7	18.5 ± 4.4	20.1 ± 4.7	b,c,d,e
D_2%_ [Gy]	41.7 ± 5.4	42.5 ± 5.3	28.7 ± 7.3	28.0 ± 6.8	b,c,d,e

The p < 0.05 for: a (3D-Pre vs 3D Post); b (3D-Pre vs RA-Pre); c (3D-Pre vs RA-Post); d (3D Post vs Ra-Pre); e (3D-Post vs RA-Post); f (RA-Pre vs RA-Post).

For OARs the results were the following: for the bladder V_40Gy_ = 41.1 ± 24.8% for 3D pre, 23.7 ± 20.1% for RA pre, 29.7 ± 18.7% for 3D post and 16.0 ± 10.7% for RA post; the mean dose for right and left femur resulted almost equivalent: 32 Gy for 3D pre, 30 Gy for 3D post and 19 Gy for RA pre and post guidelines.

The mean values of MU/Gy for 3D plans were 179.2 ± 17.2 and 180.4 ± 6.8 respectively for pre and post guidelines, while for RA plans these values were 198.6 ± 26.2 and 187.3 ± 18.3 for pre and post guidelines plans respectively.

## Discussion

This work is located inside the topic of quantifying and improving the precision and accuracy of the RT treatments with an interdisciplinary approach, as summarized by Yorke at al. in the anniversary paper on the role of medical physicists in improving geometric aspects of treatment accuracy and precision [9]. Imprecise localization of internal anatomy, tissue in-homogeneities, patient voluntary and involuntary motions, and other kind of human induced uncertainties can lead to inaccuracies much greater than the 1–2% of the usual absolute dose calibration uncertainty. In this report the dosimetric consequences of inter-observer variability in target contouring for different techniques was evaluated. The rectal tumor case was chosen as representative of challenging target definition and for its concave shape, very suitable for intensity modulated techniques.

Inter-observer variability in target volume delineation is demonstrated to be one of the major factors contributing to the global uncertainty in radiation treatment planning [7,8,10]. Accurate target delineation is extremely important to make sure that the CTV is not under-treated and to limit the dose to surrounding normal tissue. Despite the well-known consequences of geometric inaccuracy in target volume delineation [11-13], variability in target delineation has been demonstrated in several studies and for various anatomic tumor sites [13]. In the case of non-small cell lung cancer, for example, Steenbakkers *et al*. [14] reported that the size of GTV ranged from 36 cm^3^ to 129 cm^3^ (ratio 3.6, average 69 cm^3^), while van Sornsen de Koste *et al*. [15] found that the average GTV for the main tumor of a cT2N2M0 lung cancer was 13.6 cm^3^ (SD 5.2 cm^3^, median 12.3 cm^3^, range 8.3-26.9 cm^3^) as determined by 16 radiation oncologists. Concerning rectal cancer, CTV delineation presents a great variability in literature. Fuller *et al*. [7] analyzed a set of patients very similar to the ones in this study in terms of tumor stage and found a range in CTV delineation between 590 cm^3^ and 820 cm^3^; this result is comparable with our findings (CTV range between 499 cm^3^ and 994 cm^3^). The impact of the uncertainties should be evaluated again whenever a new modality of treatment delivery is introduced in the clinical practice and for RA this evaluation was already performed from other points of view [16,17]. This is particularly important since an increase in precision and conformation of dose distribution usually leads to heavier effects on dose distribution due to geometric uncertainties.

In this study we have evaluated the dosimetric impact of introduction of shared guidelines in the contouring of rectal cancer target. This project was done as part of an internal process of risk analysis in RT [18]. A total of 80 contours were generated and analysed as pre-requisite to perform the dosimetric analysis. In fact only verifying the consistency of the contouring variability with data reported in recent literature with higher populations is possible to perform the dosimetric analysis. In detail, AI was calculated pre and post guidelines coupling the contours of the various oncologists. The AI value increased of about 10%, revealing a higher homogeneity in defining the target. This result agrees with literature. Regarding rectal cancer targets, in the study of Fuller *et al*. [7] variation was analyzed volumetrically using the *conformation number* (CN, where CN = 1 equals total agreement). This research showed that a consensus atlas led to a significant increase of inter-observer agreement and CN increased from 0.58 to 0.69. Something similar was found by Myerson *et al*. [5] using *Kappa statistics* as a measure of agreement between participants: without any protocol *K* mean value was 0.49. Comparable results were found also for different sites. In a multi-institute study by Van Mourick *et al*. [19] a *conformity index* (CIvm), corresponding to the AI reported in this study, was determined (per patient and per observer couple) dividing the common volume by the encompassing volume (CIvm = 0 indicates no overlap between the two observers, whereas CIvm = 1 indicates perfect overlap). CIvm value passed from 0.3 to 0.8 with the introduction of contouring guidelines. This result is comparable with the one found by Batumalai *et al*. [20]: using a contour reference guide for the delineation of breast target, a mean *concordance index* of 0.81 was evaluated. A similar result (mean concordance index of 0.87) was reported by Struikmans *et al*. [21] for the same site. The inter-observer concordance increasing value, found in this research as well as in literature, indicates that the use of a contouring protocol may contribute to decrease inter-observer variability. Moreover, the reduction of mean CTV volumes after the introduction of guidelines (649 cm^3^ vs 595 cm^3^) can be due to the higher confidence in contouring that avoids excessively conservative contours, for example guidelines reduced the uncertainties regarding inclusion of external iliac nodes as can be seen in Figures 2 and 3. This ensures a further OAR sparing, as the irradiated volume is reduced.

Once verified the consistency of our contouring results we focused on the evaluation of the dosimetric uncertainty due to contouring observer variability for both 3DCRT and VMAT by RA techniques. Foppiano *et al*. [8] investigated the impact of inter-observer variability on rectal tumor volume and the consequences of this in DVH analysis in order to define reliable constraints for 3D conformal RT. In our series, after the introduction of guidelines the mean value of V_95%_ increased for both techniques and, at the same time, the standard deviation decreased of about 50%. In addition, improvement in PTV coverage was respectively of 3% for 3DCRT and of 8% for RA technique.

Moreover, dosimetric data showed the RA capability to reliably reproduce the dosimetric quality of conventional conformal plans, with some observable improvement such as: treatment conformality, reduction of hot spots inside target volume, reduction of OAR involvement like femurs and global reduction of HT involvement. This also confirms that normal tissue can often be better protected with IMRT and VMAT than with other conformal RT techniques, this feature was already demonstrated by other dosimetric investigations, in patients receiving pelvic radiation for anal or rectal cancer [22-25] and other anatomical regions [26-29].

While the potential of normal tissue sparing is one of the motivations behind the move towards RA for this site, thanks to the higher dose conformation, the identification of the correct target and the achievement of good target coverage remain the primary objectives and gain a still greater importance. The DVH evaluation of non-target PTVs for all optimized plans showed possible under dosages and hot-spots that made some plans unacceptable. In this setting the importance of reducing as much as possible the uncertainty in target delineation is evident. The introduction of shared guidelines is, in this context, a key intervention. In our study, for RA series, more than 50% of the plans resulted in a PTV under-dosage and this rate was reduced by more than 20% introducing guidelines. For a 3D technique this is less crucial since the absence of modulation and dose sculpting ensures acceptable target coverage despite the great PTV variability. Therefore the introduction of RA (as well as the other modulated techniques) in the clinical activity requires precise target delineation as the inverse procedure used in RA technique optimizes the dose conformation to the contoured target.

A limitation of this study is that CT was the only imaging modality used to determine the tumor target. Modern imaging techniques, such as MRI, endoscopic ultrasound, and PET could add useful information. As confirmed by different studies [12,30,31], the use of PET-CT or MRI matching may reduce inter-clinician variations, irrespective of the introduction guidelines. Another possible limitation of this study is the number of observers used for delineation, though the optimal number of observers required in such studies remains unknown. The current study had a total of ten different patients’ CT scans and four observers; this is comparable with the study by Batumalay [20], who used four observers and ten patients. Otherwise Fuller *et al*. [7] and Foppiano *et al*. [8], for example, reached similar results in target volume contouring in rectal irradiation respectively with 17 observers and 4 patients and 14 observers and only one patient’s CT scan.

## Conclusions

The introduction of guidelines reduces considerably the inter-observer variability in neo-adjuvant rectal cancer CTV delineation. In 3DCRT the minimization of contouring inter-observer variability improves the dosimetric consistency of the plans but the low dose conformation makes these changes less crucial than in modulated techniques where it is, instead, of primary importance. The introduction of shared guidelines is thus a necessary prerequisite when treating rectal cancer with modulated techniques in order to avoid severe target miss.

## Abbreviations

3DCRT: 3-Dimensional RadioTherapy; AAA: Anisotropic Analytical Algorithm; AI: Agreement Index; CT: Computed tomography; CTV: Clinical target volume; DVH: Dose volume histogram; HT: Healthy tissue; I: Intersection; IMRT: Intensity modulated radiotherapy; OAR: Organ at risk; PTV: Planning target volume; RA: RapidArc; U: Union; VMAT: Volumetric modulated arc therapy.

## Competing interest

Dr. L. Cozzi is Head of Research at Oncology Institute of Southern Switzerland and acts as a Scientific Advisor to Varian Medical Systems. The other authors declare that they have no competing interests.

## Authors’ contributions

FL, MB and PM participated in the design of the study. MB, AT, ST, FA, AG, PN, and MS carried out the data and participated in the data evaluation. GR, FA, AF, and LC performed the statistical analysis. FL, MB and GR drafted the manuscript. The definitive supervision of the paper was done by MS, LC and PM. All authors read and approved the final manuscript.
